# Nick Drake: A Troubled Mind, a Troublesome ‘Cure’

**DOI:** 10.1002/hup.2916

**Published:** 2024-12-26

**Authors:** David S. Baldwin

**Affiliations:** ^1^ Clinical Neuroscience Faculty of Medicine University of Southampton Southampton UK

The British Association for Psychopharmacology (BAP) concluded its Fiftieth Anniversary Summer Meeting in July 2024 with a plenary symposium in which BAP stalwarts glanced back and looked forward, to identify achievements in psychopharmacology since 1999 and consider likely developments in neuroscience before 2049. There was much to ponder, whilst travelling home from the University of Birmingham: and my dream‐like musings continued that same evening, when the BBC Radio 3 Promenade (‘Prom’) concert broadcast was devoted solely to ‘An Orchestral Celebration’ of the music of Nick Drake, who died when aged only twenty‐six, almost fifty years before, in November 1974.

Born in Rangoon, Burma (now Yangon, Myanmar), to expatriate Britons—his father worked as a Civil Engineer, his mother came from a senior Indian Civil Service family—Nicholas Rodney Drake moved with his family to the UK as a toddler, went to a preparatory boarding school, then boarded at Marlborough College, before somewhat unexpectedly scraping into Fitzwilliam College Cambridge in 1967, to read English Literature. But he left Cambridge two years later, nine months before graduation, against the advice of his tutors and family, to further his musical career: his first album, the wistful and pastoral *Five Leaves Left* had been released a few months earlier. A strike‐delayed second album, *Bryter Later*, sometimes considered to be ensemble‐heavy, appeared in March 1971. Both were critically acclaimed by music journalists and other musicians, but neither gained much traction with the buying public. His third album, the desolate and intermittently agitated *Pink Moon*, was released in February 1972, but probably sold less than its predecessors.

Characteristically reticent and often seeming aloof, he became steadily more isolated and withdrawn, possibly linked to or worsened by regular smoking of cannabis, which he started before University. He declared himself increasingly reluctant to play the live concerts which could probably have led to wider recognition and greater sales. Although he was an astonishingly adept, technically innovative guitarist and widely respected lyricist, it was more challenging for him to synchronise playing with singing. He sought company but was uncommunicative whilst in it. He neglected himself, seemed erratic and unpredictable, and sometimes had observable difficulty in sequencing tasks, such as making tea. Increasing parental concern prompted admission to the local inpatient psychiatric unit, where after a few weeks the thoughtful, compassionate consultant psychiatrist suggested their 23‐year old son might suffer from simple‐type schizophrenia.

Psychiatric diagnoses can be unreliable, and treatment plans are often contested. Drake himself stated that taking medication was ‘against my principles’. The well‐meaning social worker attached to the local unit indicated that psychotropic medication was not as beneficial as psychotherapy, and recommended referral to a Laingian psychotherapist. By contrast, a regional specialist consultant psychiatrist recommended electroconvulsive therapy (ECT). Subsequently, an eminent London teaching hospital consultant, seeing Drake on a private basis, preferred the diagnosis of depressive illness and recommended combination treatment with amitriptyline, trifluoperazine, orphenadrine and diazepam. Drake did not attend an offered appointment with the Laing‐founded Philadelphia Association. ECT was administered just once. Periods of medication adherence were accompanied by some beneficial effects, including the desire to write new material, but treatments and improvements were not sustained. He became increasingly despondent, took a large intentional overdose of diazepam (thirty‐six tablets) in February 1973, and died in autumn of the following year after a substantial overdose (approximately sixty tablets) of amitriptyline: the coroner's inquest recorded a verdict of suicide ‘when suffering from a depressive illness’.

On balance, the multiple insightful observations of friends and peers featured within the forensically‐detailed biography (Jack 2023) align with some important features (including negative symptoms and disorganisation) indicating a probable diagnosis of schizophrenia. His father believed this to be the case and had joined the National Schizophrenia Fellowship for support and advice: the always supportive, loving family were troubled by a sense of bewildered helplessness. They had not been made aware that amitriptyline could be fatal when taken in even small overdoses. A close friend from his University days, who subsequently became a psychiatrist, felt that regardless of diagnosis, psychotropic medication alone would have been insufficient to result in much improvement.

But regardless of favoured diagnosis, the inpatient and outpatient clinical care which Drake received was unable to arrest an inexorable decline. Current mental health services—such as early intervention in psychosis teams—would strive to provide a case‐managed, community‐based, multi‐disciplinary approach including assertive outreach, advice against returning to cannabis smoking, psychotropic medication optimisation (perhaps involving long‐acting injectable antipsychotic treatment), psychological treatment, recovery‐based activities and family involvement and support. With such provisions at the time, the outcome for this troubled young man may well have been different.

An awareness of his struggle with personal mental health problems seems important when considering the curtailed output of Nick Drake, but acknowledgement of the artistic legacy rests on his crafted musicianship, the high esteem accorded his work by his peers, and the appreciation of a devoted, increasing audience. By the time of his death, the three albums may together have sold fewer than four thousand copies. But even in the punk era, when most folk‐rock singer‐songwriters were viewed suspiciously, his autodidactic, uncompromising talent was recognised and understood. His reputation has grown steadily, and record sales have increased over each decade of the last fifty years: the promotion for the BBC3 Prom rightly stated that he has reached a ‘new, cross‐generational audience, many of them beguiled by his fragility and fatalism, his music's mingling of the outwardly simple and the inwardly complex’.

## Conflicts of Interest

The author declares no conflicts of interest.

## Data Availability

The author has nothing to report.
